# Shape Memory Polyurethanes Based on Zwitterionic Hard Segments

**DOI:** 10.3390/polym9100465

**Published:** 2017-09-21

**Authors:** Shuqin Fu, Huanhuan Ren, Zaochuan Ge, Haitao Zhuo, Shaojun Chen

**Affiliations:** 1 Shenzhen Key Laboratory of Functional Polymer, College of Chemistry and Environmental Engineering, Shenzhen University, Shenzhen 518060, China; fusq@szu.edu.cn; 2 Key Laboratory of Optoelectronic Devices and Systems of Ministry of Education and Guangdong Province, College of Optoelectronic Engineering, Shenzhen University, Shenzhen 518060, China; 3 Guangdong Research Center for Interfacial Engineering of Functional Materials, Shenzhen Key Laboratory of Polymer Science and Technology, Shenzhen Key Laboratory of Special Functional Materials, Nanshan District Key Lab for Biopolymers and Safety Evaluation, College of Materials Science and Engineering, Shenzhen University, Shenzhen 518060, China; rhh1171849722@163.com (H.R.); gezc@szu.edu.cn (Z.G.)

**Keywords:** shape memory, polyurethane, *N*-methyldiethanolamine, zwitterionic polymer, biocompatibility

## Abstract

This work aimed at elucidating the influence of zwitterionic hard segments on the structures and properties of shape memory polyurethanes (SMPUs). A series of zwitterionic SMPUs was successfully prepared with *N*-methyldiethanolamine (MDEA), 1,3-propanesultone (1,3-PS), 1,6-hexamethylene diisocyanate (HDI) and polyethylene glycol (PEG6000). The influence of MDEA-PS-based zwitterionic hard segment on structure, morphology, thermal property, shape memory property and cytocompatibility were systematically investigated. The results demonstrated that the PEG-based zwitterionic SMPUs (PEG-ZSMPUs) formed phase separation structure consisting of crystalline soft phase and amorphous hard phase. The MDEA-PS zwitterionic segments showed a tendency to form ionic clusters in hard segments, which served as reinforced net points. Shape memory analysis showed that zwitterionic PEG-ZSMPUs containing a high content of zwitterionic segments had thermal-induced shape memory effects. Finally, cytotoxic assays demonstrated that MDEA-PS zwitterionic segment improved the biocompatibility of PEG-ZSMPUs. The zwitterionic PEG-ZSMPUs could thus have a promising application in smart biomedical fields.

## 1. Introduction

Polyurethanes are a general segmented copolymer comprising of hard and soft segments [1]. In the past several decades, polyurethanes possessing shape memory effects (SMEs) have been widely studied due to their capabilities to fix temporary shapes upon cooling and recover to the original shapes upon heating [2,3]. So far, many kinds of polyurethanes have been found to show various SMEs, including thermal-induced SMEs, moisture-sensitive SMEs, and multi-SMEs [4]. In the shape memory polyurethanes (SMPUs), the hard segments generally determine permanent shapes and serve as physical net points, while the soft segments control the fixing and recovering of temporary shapes, and serve as switches [5,6]. Adjusting the structure and content of either hard or soft segments can cause SMPUs to have quite different morphologies and properties. Thus, various kinds of soft and hard segments have been used to control the shape memory properties [7], and in order to meet such complex requirements in the practical applications, SMPUs with more functionalities have been rapidly developed. More attentions are drawn in particular to the recent developed multi-functional SMPUs [8,9,10].

Generally, the design and synthesis of multifunctional SMPUs require key functional monomers, which can be further functionalized or triggered to have various properties, such as biocompatibility, electrical conductivity, and self-healing properties [11,12,13]. The biocompatibility is the key factor influencing the biomedical applications of SMPUs. Polyethylene glycol (PEG), which has good biocompatibility, is a good candidate of soft segment for the synthesis of biomedical SMPUs [12]. Bonfil et al. synthesized a series of polyurethane films that were composed of PEG and castor oil by using the one-shot polymerization method [14]. They have shown that not only do PEG-based SMPUs have excellent thermal-induced shape memory effects, but they also have some interesting properties, such as water-triggered shape memory effect. In addition, our recent investigation has found that a higher content of hard segments might influence the biocompatibility of SMPUs [12]. Therefore, it is necessary to further modify the hard segments of SMPUs in order to meet the need in biocompatibility. 

Recently, *N*-methyldiethanolamine (MDEA), which contains tertiary amine, has been used in the design of stimulus-responsive polyurethanes to have temperature and pH dual-responsive characteristics [15]. It is well known that the tertiary amine in the MDEA segment can be protonated to become ionic form [16,17]. The polyurethane-containing MDEA segment can thus be ionized with acetic acid or 1,3-propanesulonate (PS) to zwitterionic polyurethanes, which has been widely reported to have a good biocompatibility [18,19]. Our recent work has confirmed that the zwitterionic polyurethanes based on HDI and MDEA showed not only moisture-sensitive SMEs, but also had good biocompatibility and antibacterial properties [20,21]. To date, it is known that the thermal-induced SME is greatly influenced by the MDEA-PS-based zwitterionic segments; however, its influence when served as hard segments is still unclear. 

Aimed at designing SMPUs to have both good biocompatibility and shape memory properties, this work designed a new kind of zwitterionic SMPUs by incorporating PEG soft segments and MDEA based zwitterionic hard segments. Based on our previous communications on PEG-based SMPUs, zwitterionic SMPUs, and amide-containing SMPUs [22], a series of PEG-based zwitterionic SMPUs (denoted PEG-ZSMPUs) were synthesized from MDEA, HDI, PEG and 1,3-PS by adjusting the molar ratio of 1,3-PS and MDEA. The influence of zwitterionic hard segments on the structure, morphology, thermal properties, shape memory properties and cytocompatiblity of PEG-ZSMPUs were then systematically studied.

## 2. Experimental

### 2.1. Materials

Dibutyltindilaurate (DBTDL; analytical reagent), 1,6-hexamethylene diisocyanate (HDI; analytical reagent), *N*-methyldiethanolamine (MDEA; analytical reagent), polyethylene glycol (number-average molecular weight of 6000, PEG6000; premium grade), 1,3-propanesultone (1,3-PS; analytical reagent), and dimethylformamide (DMF; analytical reagent) were purchased from Aladdin Chemical Reagent Co. Ltd. (Shanghai, China), and used without further purification.

### 2.2. Synthesis of PEG-ZSMPUs

**Scheme 1:**
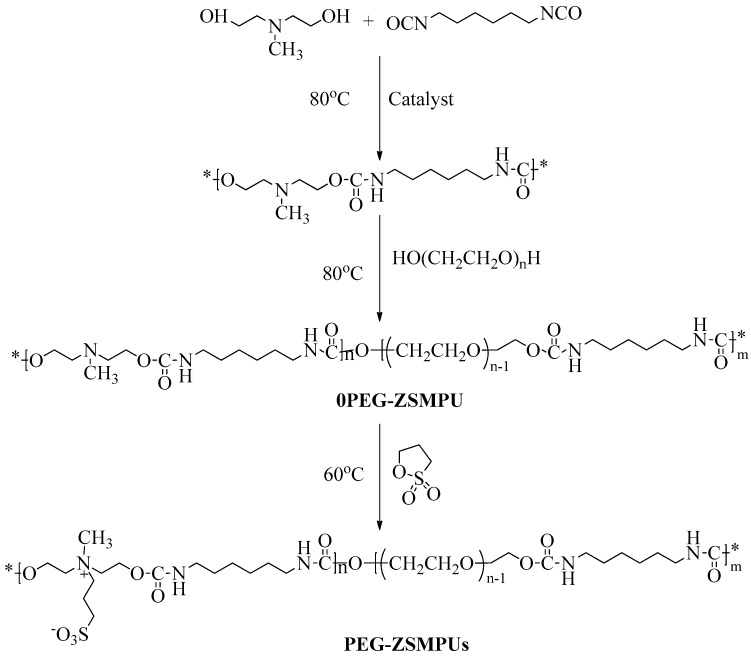
The synthesis route of PEG-ZSMPUs.

A series of PEG-ZSMPUs were synthesized from different molar ratios of 1,3-PS and MDEA by polyurethane polymerization and ring-opening reactions according to the composition shown in Table 1. The procedure in preparation of PEG-ZSMPUs is presented in Scheme 1. The preparation was conducted in an argon-filled and a mechanically stirred 250-mL three-neck flask. First, chemicals including MDEA (3.32 g, 0.0279 mol), HDI (5.01 g, 0.0297 mol), and DMF (10 mL) were mixed at 60 °C. After purging and stirring for 15 min, DBTDL catalyst (0.15 wt % of polyurethane) was added to the mixture [22]. When the temperature was increased to 80 °C under vigorous stirring, the reaction mixture became violent and its viscosity was significantly increased. At 1.5 h into the reaction, DMF was added to the reaction to control its viscosity, followed by PEG6000 (11.67 g, 0.0019 mol), and then left for an additional 3 h. After that, 1,3-PS (3.40 g, 0.0278 mol) was added, and the reaction was conducted hermetically at 75 °C for 12 h. The molar ratios of 1,3-PS and MDEA were adjusted according to composition presented in Table 1. Finally, DMF was evaporated from the resulting SMPU/DMF solution in a Teflon pan and in a vacuum at 80 °C for 12 h. The target zwitterionic SMPU were coded “#PEG-ZSMPU”, where “#” is the number representing the molar ratio of 1,3-PS and MDEA. 

### 2.3. Characterization of PEG-ZSMPUs

Fourier transform-infrared spectroscopy spectra (FT-IR, Thermo Nicolet FT-IR 6700, Nicolet, Madison, WI, USA) of PEG-ZSMPUs were scanned from 4000 to 400 cm^−1^ wavelengths. The spectra were the average of 32 scans at a resolution of 4 cm^−1^.

The ^1^H-nuclear magnetic resonance spectra (^1^H-NMR, Avance III 400MHz, Bruker, Switzerland) were recorded using DMSO-*d*_6_ as the solvent and tetramethylsilane (TMS) as the internal standard. 

Elemental analysis (EA, Vario EL III, Elementar Analysen-systeme GmbH, Hanau, Germany) was conducted to determine the weight percentage of S, N, C, and H of PEG-ZSMPUs. 

The XPS analysis (XPS, VG multilab2000, Thermo Electron Corporation, Waltham, MA, USA) was carried out using anode voltage and current of 15 kV and 10 mA, respectively. The elemental composition was determined on the basis of peak areas and sensitivity factor from C_1s_, N_1s_, O_1s_, and S_2p_ peaks using advantage software. All of the binding energy values were confirmed with reference to carbon (C_1s_ = 284.6 eV).

X-ray diffractometry (XRD, D8 Advance, Bruker, Billerica, Germany) was performed to measure the crystallization of PEG-ZSMPUs. The XRD data were recorded in Bragg’s angle 2θ ranging from 5 to 90° with a scanning step size of 0.02°/s. 

The crystalline morphology and polarizing optical patterns of PEG-ZSMPUs were observed at 25 °C by using a polarized optical microscope (POM, MP41, Mshot, Guangzhou, China), which was equipped with a hot stage (Mettler Toledo FP90 Central Processor & FP82 Hot Stage), and a camera was used to observe and record the strain recovery behaviors upon heating and cooling at a rate of 2 °C/min. 

Atomic force microscopy (AFM, Nanonavi E-Sweep, SII Nanotechnology Instrument, SEIKO Co. Ltd, Tokyo, Japan) was used in tapping mode for morphological characterization of PEG-ZSMPUs. PEG-ZSMPUs were dissolved in DMF at a concentration of 5 mg/mL, which was then spin-coated on oxidized silicon substratesat 400 rpm for 10 s, followed by 4000 rpm for 60 s. The resulting spin-coated films were kept in a vacuumed oven at 50 °C for 48 h to allow for the evaporation of the solvent. 

The morphology of PEG-ZSMPUs was examined using scanning electron microscopy (SEM, NGB4-DXS-10AC, Hitachi, Hitachi, Japan). Prior to SEM scanning, PEG-ZSMPUs were coated with a thin layer of gold. All of the measurements were repeated three times.

Differential scanning calorimeter (DSC, Q200, TA universal, New Castle, DE, USA) was conducted in a purge gas, nitrogen to measure the glass-transition temperature (*T*_g_) and the melting temperature (*T*_m_) of PEG-ZSMPUs. PEG-ZSMPUs were first heated from −50 to 150 °C at a heating rate of 10 °C/min. The temperature was kept constant at 150 °C for 1 min before cooling down to −50 °C at a rate of 10 °C·min^−1^. After that, the samples were reheated and scanned from −50 to 150 °C, at which the *T*_g_ and *T*_m_ were recorded. 

Thermogravimetric analysis (TGA) and derivative thermogravimetric analysis (DTG) were recorded with Q50 (TA universal, New Castle, DE, USA) at a heating rate of 10 °C/min from 100 to 600 °C in platinum crucibles under nitrogen flow (60 mL/min), and the sample weight of approximately 10 mg.

Dynamic mechanical analysis (DMA, Q200, TA universal, USA) was determined in tensile mode with tension clamps under nitrogen at a heating rate of 3 °C/min from −50 to 180 °C. The rectangle specimens for DMA tests were prepared by film casting with approximate dimensions of 20 mm × 6 mm × 0.4 mm. Specimens were determined under 1.0 Hz.

Shape-memory behaviours were also determined qualitatively with thermo-mechanical analysis using a TA Instruments DMA800 (TA universal, New Castle, DE, USA) (using tension clamps in controlled force mode). All of the samples were dried at 100 °C in vacuo for 24 h and cut into rectangular pieces of approximately 10 mm × 2.0 mm × 0.5 mm. The detailed test setup for the shape-memory cycles is provided herein: (1) heating to approx. 55 °C, followed by equilibration for 20 min; (2) uniaxial stretching to strain (ε_load_) by ramping the force from 0.001 N to 1 N at a rate of 0.25 N/min, followed by equilibration for 3 min; (3) fixing the strain (ε) about 100% by rapidly cooling to approx. 20 °C with *q* = −10 °C/min, followed by equilibration for 10 min; (4) unloading an external force 0 N at a rate of 0.25 N/min; (5) reheating to approx. 55 °C at a rate of 4 °C/min, followed by equilibration for 40 min; and finally, the recovery strain (ε_rec_) was recorded. 

The cytocompatibility of PEG-ZSMPUs was evaluated with regard to cell viability. Murine macrophage RAW264.7 cells (Shanghai Institute of Biochemistry and Cell Biology, Shanghai, China) were cultured at 37 °C in RPMI-1640 medium supplemented with 10% fetal bovine serum, 100 IU·mL^−1^ penicillin, and 100 μg·mL^−1^ streptomycin in a humidified atmosphere with 5% CO_2_. The cytotoxic effects of PEG-ZSMPUs were assessed with regard to cell viability of RAW264.7 cells, measured by tetrazolium salt (WST-8) based colorimetric assay according to Cell Counting Kit 8 (Dojindo, Kumamoto, Japan) [23]. The morphology cells grown on PEG-ZSMPUs films were observed by optical microscopy (ZEISS Axio Imager A1 m).

## 3. Results and Discussion

### 3.1. Structures of PEG-ZSMPUs

**Figure 1:**
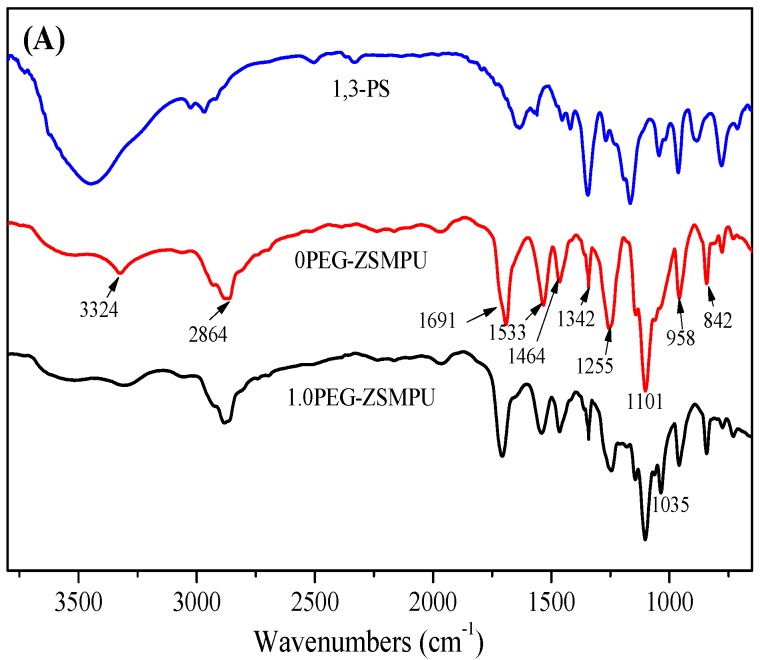

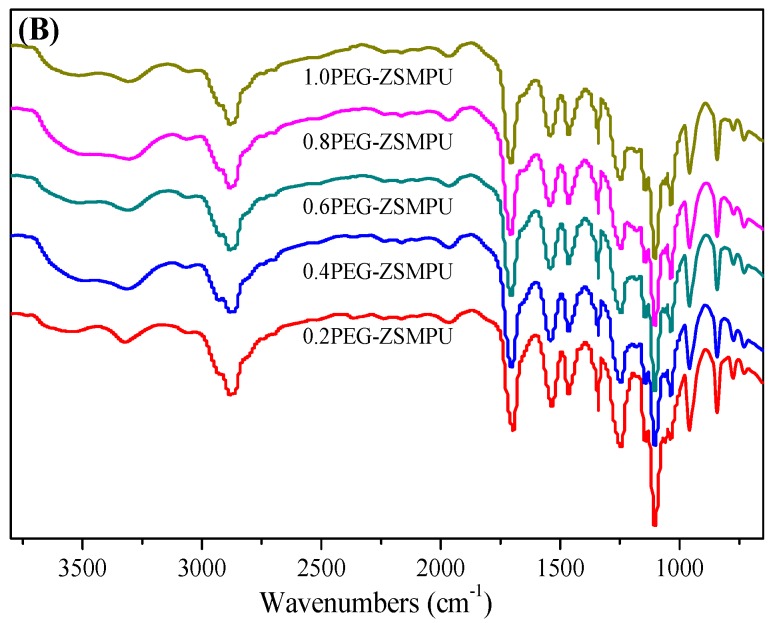
Fourier transform-infrared spectroscopy spectra (FT-IR) spectra of samples, (**A**) for comparisions of 1,3-PS, 0PEG-ZSMPU and1PEG-ZSMPU; (**B**) for PEG-ZSMPUs with different zwitterionic hard segments.

**Figure 2:**
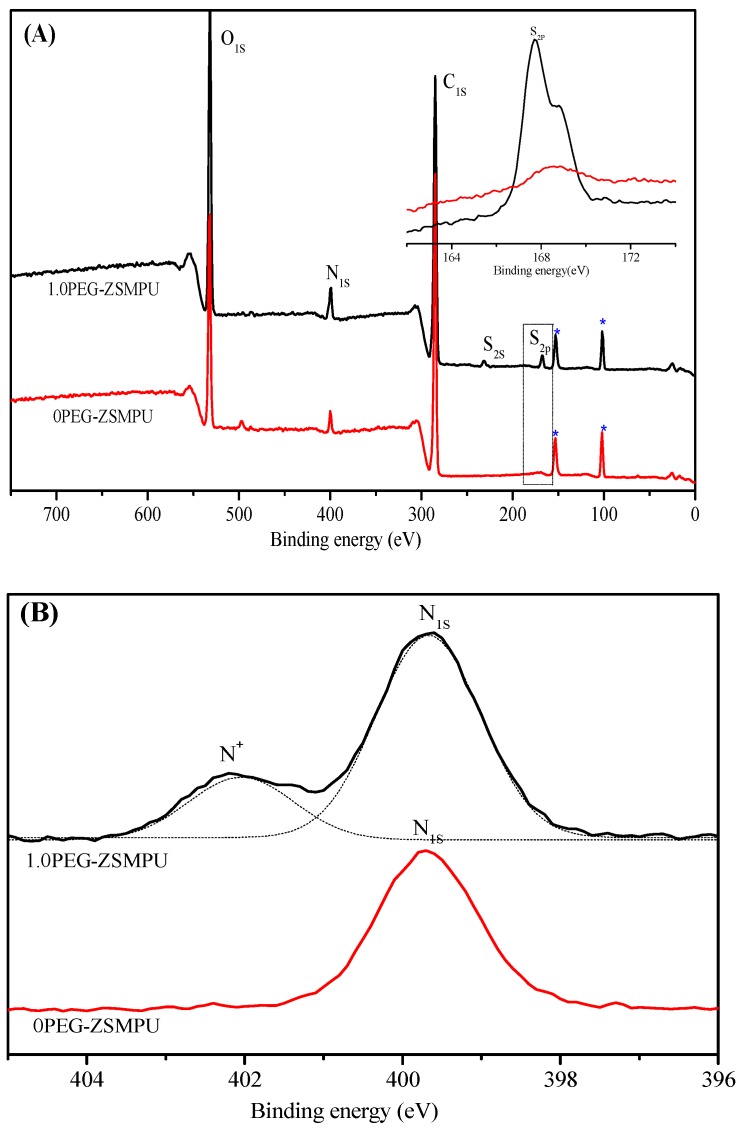
X-ray photoelectron spectroscopy (XPS) and N_1s_ spectra of 0PEG-ZSMPU and 1.0PEG-ZSMPU. ((**A**) Full spectra; (**B**) N_1s_ spectra).

To verify the success in preparation of PEG-ZSMPUs, structures of PEG-ZSMPUs were investigated by FT-IR (Figure 1) and ^1^H-NMR (Figure S1, see supporting information). The FT-IR spectrum of 1.0PEG-ZSMPU exhibited absorbance at 3324, 1691 and 1533 cm^−1^, which could be assigned to N–H and C=O stretching vibration and C–N deformation vibration of urethane groups, respectively. The low stretching vibration frequency of C=O group suggested that strong hydrogen bonds were formed among both soft and hard segments. When comparing with that of 0PEG-ZSMPU (Figure 1A), the FT-IR spectrum of 1.0PEG-ZSMPU showed an additional peak at 1035 cm^−1^, which could be attributed to the absorption of S=O group of sulfonic acid group (Figure 1). In addition, intensity of the peak enhanced with increasing molar ratios of 1,3-PS and MDEA in PEG-ZSMPUs (Figure 1B). Moreover, the absorbance of isocyanate groups at 2272 cm^−1^ was not observed in HDI-MDEA-PS hard segment. A comparison of ^1^H-NMR spectra of 0PEG-ZSMPU and 0.8PEG-ZSMPU further confirmed that chemical shift at 3.10 ppm (Figure S1e) could be assigned to proton signals of –N^+^–CH_3_. Proton signals corresponding to the –CH_2_– of sulfonic acid were observed from 0.8PEG-ZSMPU at 1.70, 2.77, and 3.67 ppm (Figure S1c,d,f, respectively). When comparing with the ^1^H-NMR spectrum of 0PEG-ZSMPU, chemical shifts in reference to –CH_2_– of MDEA were observed in 0.8PEG-ZSMPU (from 3.98 ppm in 0PEG-ZSMPU to 4.16, and 4.36 ppm in 0.8PEG-ZSMPU). In addition, chemical shift associated with the N–H of 0.8PEG-ZSMPU were also found (from 7.0 ppm in 0PEG-ZSMPU to 7.2–7.4 ppm in 0.8PEG-ZSMPU). The elemental composition of PEG-ZSMPUs was determined by using an elementalanalyzer and found that the actual content of elemental S in PEG-ZSMPUs was close to its theoretical weight percentage (Table S1, see supporting information). Additionally, X-ray photoelectron spectroscopy (XPS) of 0PEG-ZSMPU and 1.0PEG-ZSMPU further confirmed the presences of S_2s_ (binding energy, 230 eV) and S_2p_ (binding energy, 168 eV) orbitals in 1.0PEG-ZSMPU, which were absent in 0PEG-ZSMPU (Figure 2A). The N_1s_ spectrum of 1PEG-ZSMPU showed two peaks at 402.2 and 399.6 eV, indicating the presences of ammonium nitrogen in MDEA-PS segment and nitrogen of either urethane or un-reacted MDEA monomer, respectively (Figure 2B). These results confirmed that the designed PEG-ZSMPUs were successfully prepared.

### 3.2. Morphology of PEG-ZSMPUs

**Figure 3:**
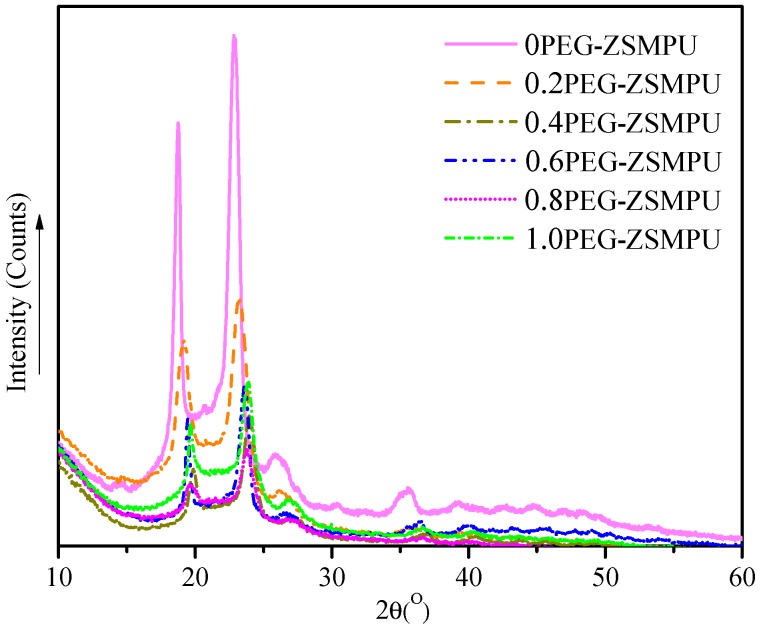
X-ray diffractometry (XRD) curves for PEG-ZSMPUs.

**Figure 4:**
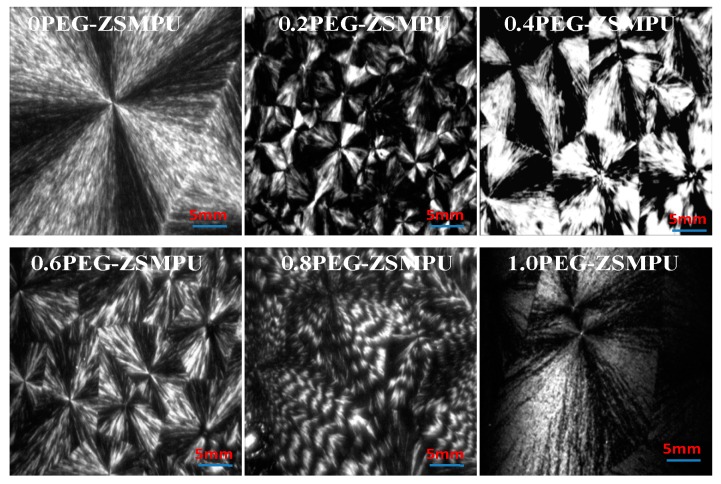
Polarized optical microscope (POM) images (magnified 100 times) of PEG-ZSMPUs.

The morphology of PEG-ZSMPUs was investigated by XRD, POM, AFM and SEM. Figure 3 shows the XRD curves of PEG-ZSMPUs with different molar ratios of 1,3-PS and MDEA. The XRD patterns demonstrated that PEG-ZSMPUs had two obvious crystallization peaks near 2θ = 18.7° and 22.8°, indicating that the crystal has high integrity with large size. Moreover, intensities of crystallization peaks decreased first and then increased, with the increased molar ratio of 1,3-PS and MDEA. As could be observed, the intensities of crystallization peaks of 0.4PEG-ZSMPU was lower than that of 0.6PEG-ZSMPU, while the intensities of crystallization peaks of 1.0PEG-ZSMPU was lower than that of 0PEG-ZSMPU. The reason is that the aggregation of zwitterionic hard segment firstly promotes the crystallization of PEG soft phase, whereas too much higher content of hard segments result in the soft-hard phase mixing, destroying the crystallization. Furthermore, it was observed that positions of the peak shifted to higher values when the molar ratio of 1,3-PS and MDEA increased. The peak positions of 0PEG-ZSMPU, 0.2PEG-ZSMPU, 0.4PEG-ZSMPU and 0.6PEG-ZSMPU shifted to higher peak values, while those of 0.8PEG-ZSMPU and 1.0PEG-ZSMPU shifted to lower peak values, which however were higher than that of 0PEG-ZSMPU. This indicated that the crystallization ability of PEG-ZSMPUs decreased and then enhanced, which may be ascribed to the grafting of 1,3-PS on to the main chain of PEG-ZSMPUs as well as the formation of branched chain that could destroy the regularity of molecular chain so that the crystallization ability of PEG-ZSMPUs was weaken. The POM image provided a direct visual-proof of the crystallization of PEG-ZSMPUs. As shown in Figure 4, PEG-ZSMPUs had many “+” polarizing optical patterns of the spherical crystals, indicating that PEG-ZSMPUs have good crystallinity. Moreover, the crystallization rate of PEG-ZSMPUs decreased and then enhanced, with the increasing 1,3-PS content, which was consistent with the result from XRD analysis.

**Figure 5:**
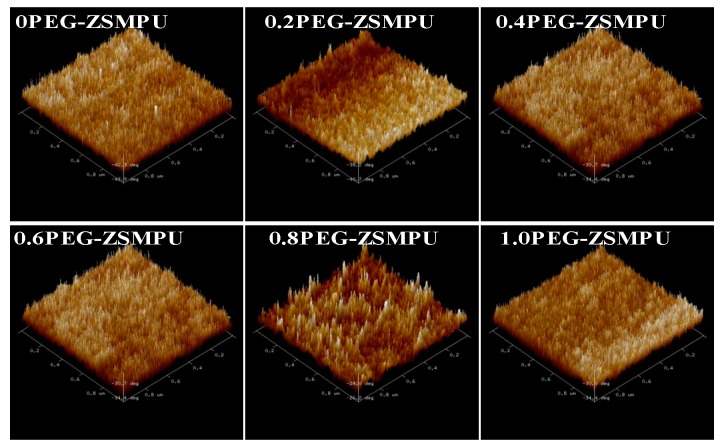
Atomic force microscopy (AFM) images (3D phase) of PEG-ZSMPUs.

**Figure 6:**
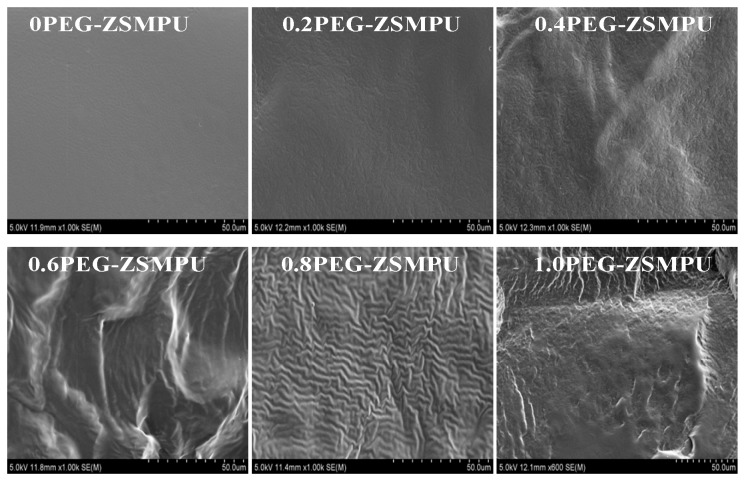
Scanning electron microscopy (SEM) images of PEG-ZSMPUs.

AFM technology provides a persuasive evidenceto visually study the morphology of PEG-ZSMPUs. The AFM images of PEG-ZSMPUs showed a phase separation structure comprising of hard and soft phases. The 3D phase AFM images showed some clear cuspidal outshoots (Figure 5), which may be due to HDI-MDEA aggregates and ionic bond serving as hard phase and some pot holes resulted from HDI-PEG6000 soft phase. The result shown in Figure 5 demonstrated that the amorphous hard phase (HDI-MDEA) was separated by soft phase and divided into small hard domains. The hard domain increased with increasing molar ratio of 1,3-PS and MDEA from 0.2 to 0.8 (Figure 5). When the molar ratio of 1,3-PS and MDEA was greater than 0.8, the hard phase of 1PEG-ZSMPU became continuous with the soft phase embedded in it (Figure 5). This aggregation of zwitterionic polymers has been widely investigated previously [24]. The surface morphology of PEG-ZSMPUs was systematically investigated. Figure 6 displays SEM images of PEG-ZSMPUs films with different molar ratio of 1,3-PS and MDEA. The SEM images showed that 0PEG-ZSMPU had a smooth surface without any holes, whereas those of 0.2PEG-ZSMPU, 0.4PEG-ZSMPU, 0.6PEG-ZSMPU and 0.8PEG-ZSMPU were rough and appeared to have a series of bending wave patterns. As observed in 0.2PEG-ZSMPUs, 0.4PEG-ZSMPUs, 0.6PEG-ZSMPUs and 0.8PEG-ZSMPU, numbers of bending waves increased with the increase of molar ratio of 1,3-PS and MDEA (Figure 6). Results from the fracture surface of PEG-ZSMPUs demonstrated that they had typical ductile fractures. However, the fracture surface of 1.0PEG-ZSMPU was rough and irregular multilayer, which indicated that it was the brittle fracture. These results indicated that the increased molar ratios of 1,3-PS and MDEA weakened the toughness but enhanced the brittleness of PEG-ZSMPUs. 

### 3.3. Thermal Properties of PEG-ZSMPUs

**Figure 7:**
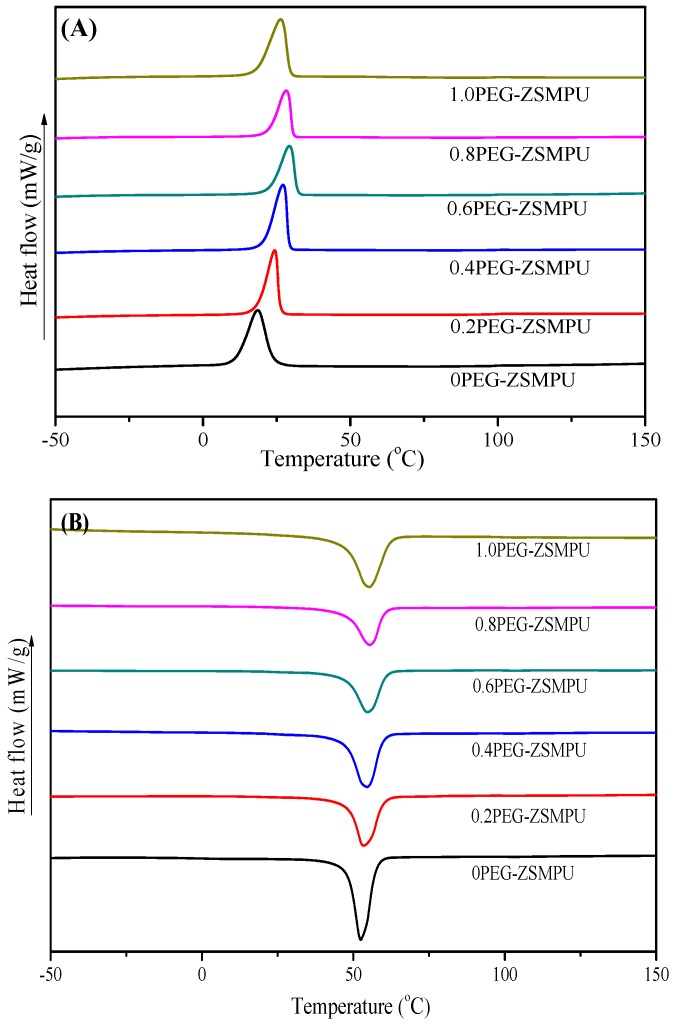
Differential scanning calorimeter (DSC) curves of PEG-ZSMPUs: (**A**) the cooling curves and (**B**) the second DSC heating curves.

**Figure 8:**
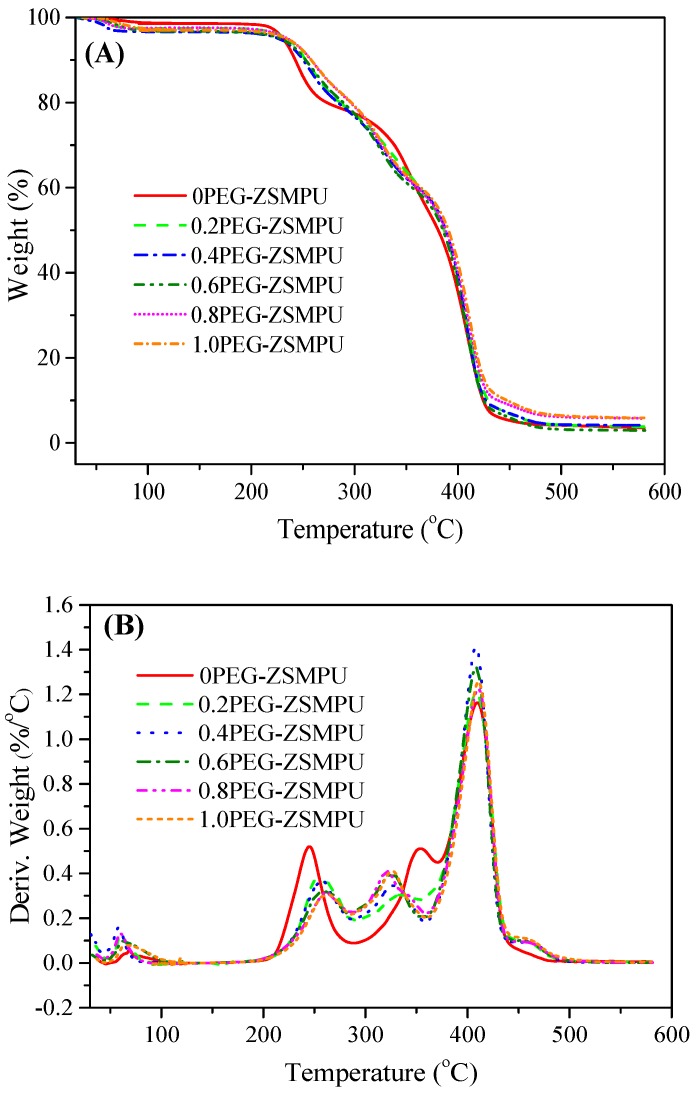
Thermogravimetric analysis (TGA curves of PEG-ZSMPUs: (**A**) TG curves, and (**B**) DTG curves).

The thermal properties of PEG-ZSMPUs, synthesized from different molar ratios of 1,3-PS and MDEA, were characterized by using DSC and TG. Figure 7 shows the DSC curves of PEG-ZSMPUs obtained from the second heating and cooling scanning process. The second DSC heating curves demonstrated that PEG-ZSMPUs had a crystalline phase at the low temperature range, which could be ascribed to PEG crystalline soft phase (Figure 7B). The zwitterions tended to improve the crystallization temperature (*T*_c_) of the soft phase. The *T*_c_ of PEG-ZSMPUs increased with the increase of molar ratios of 1,3-PS and MDEA, which could be due to an increase of ionic interactions (Figure 7A). The influence of ionic interaction was also shown on the TG curves and DTG curves (Figure 8A), demonstrating that PEG-ZSMPUs had higher decomposition temperatures when compared with that of their precursor (0PEG-ZSMPU). The maximum degradation temperatures of PEG-ZSMPUs, defined by the temperature at which the maximum decomposition rate occurs, were higher than that of their precursor in the first stage (i.e., the maximum decomposition rate occurs a temperature of 256 °C in 1.0PEG-ZSMPU, compared with that of 248 °C in 0PEG-ZSMPU) (Figure 8B). TG curves further demonstrated that residues of PEG-ZSMPUs above 500 °C enhanced with increasing molar ratios of 1,3-PS and MDEA, implying that MDEA-PS zwitterionic hard segments had a good thermal-stability. Therefore, the MDEA-PS segment had a tendency to enhance the thermal-stability of PEG-ZSMPUs.

### 3.4. Shape Memory Properties of PEG-ZSMPUs

**Figure 9:**
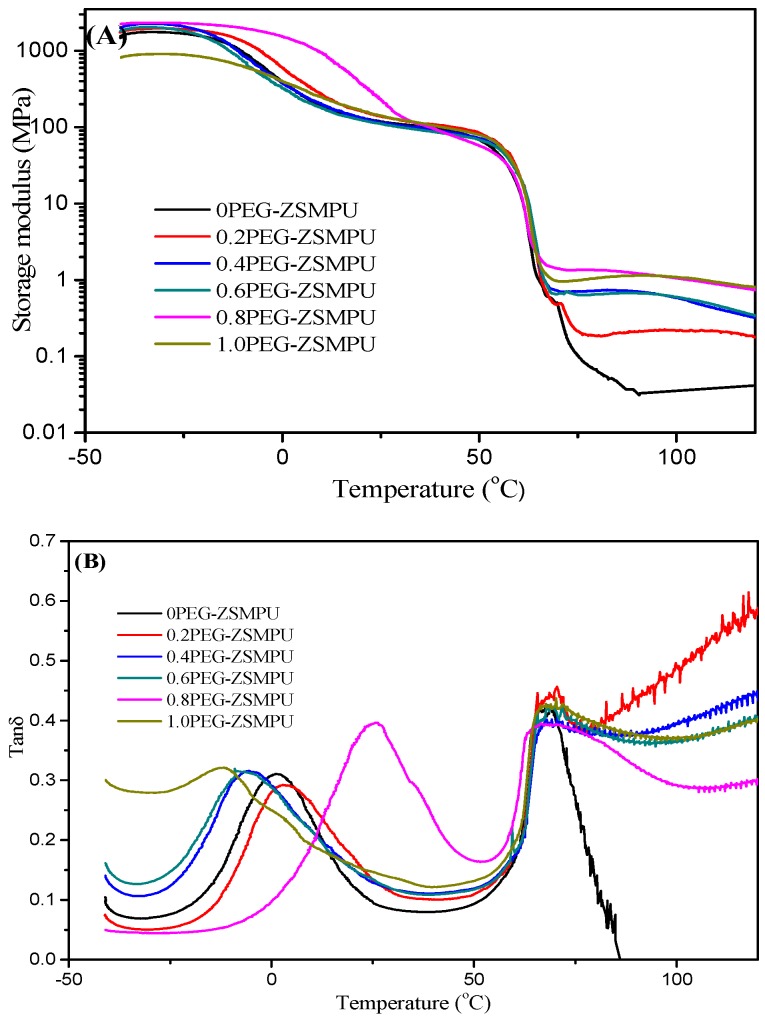
The Dynamic mechanical analysis (DMA) curves of PEG-ZSMPUs (**A**) storage modulus and (**B**) tan δ curves.

The zwitterions can affect the dynamic mechanical properties of PEG-ZSMPUs. Figure 9 shows DMA curves of PEG-ZSMPUs with different molar ratios of 1,3-PS and MDEA, representing phase transitions of PEG-ZSMPUs. In addition, tan δ curves demonstrated that the PEG-ZSMPUs went about glass and crystal melting transitions at a low temperature range (e.g., below 100 °C). The storage modulus (*E*’) curves showed that the *E*’ of PEG-ZSMPUs changed slightly in a glassy state. During the crystal melting transition process, a sharp decrease in the modulus occurred in all of the PEG-ZSMPU samples. At the rubber state, the rubber modulus increased with the increase of molar ratios, indicating the reinforcement of zwitterionic hard segments. The results showed that grafting of the 1,3-PS onto the main chain of 0PEG-ZSMPU influenced main chain’s mobility. It is possible that the degradation and creep relaxation of sulfobetaine side chain may cause glass transition of the polymer to become wider when heated. Moreover, the elevated molar ratio of 1,3-PS may break the hydrogen bond among the hard segments in PEG-ZSMPUs. While the higher modulus ratios between the glassy and rubber modulus were found in PEG-ZSMPUs, their ratios increased with the increasing ratios of 1,3-PS and MDEA. These results suggested that PEG-ZSMPUs with a higher ratio of 1,3-PS and MDEA might show thermally induced SMEs, e.g., in the 1.0PEG-ZSMPUs. 

**Figure 10:**
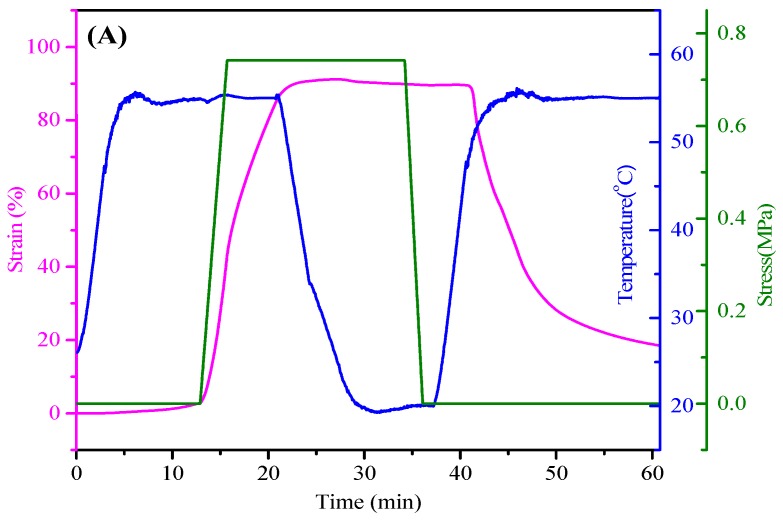

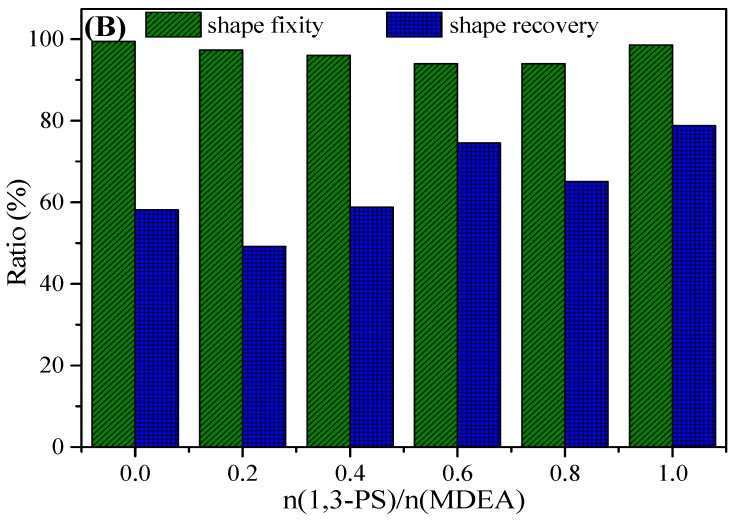
Thermal-induced shape memory effects (SMEs) of PEG-ZSMPUs: (**A**) shaped memory curves of sample 1.0PEG-ZSMPU and (**B**) shape recovery and shape fixity of PEG-ZSMPUs with various molar ratio of 1,3-PS/MDEA.

Thermal-induced SMEs of PEG-ZSMPUs were analyzed by thermal-mechanical analysis using a TA Instrument DMA Q200. The results shown in Figure 10A demonstrated that 1.0PEG-ZSMPU could be deformed to nearly 90% strain when it was heated to 55 °C; and, after being cooled down to a temperature below 20 °C, more than 94% strain could be fixed. When the sample was reheated to 55 °C, nearly 79% of the deformed strain was recovered. Although all of the samples showed good shape fixation, those without or less zwitterionic segment (e.g., samples 0PEG-ZSMPU and 0.2PEG-ZSMPU) showed a very low shape recovery. These demonstrated that the zwitterionic hard segment could improve the shape recovery by promoting the aggregation of the hard segment to serve as physical netpoints. 

### 3.5. CytocompatibilityAnalysis of PEG-ZSMPUs

**Figure 11:**
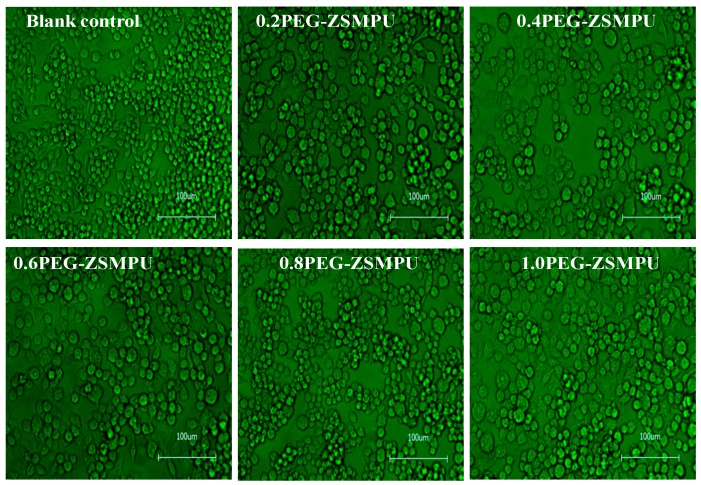
The inverted micrographs of RAW264.7 cells after incubating with sample PEG-ZSMPUs for 48 h.

In vitro biocompatibility of PEG-ZSMPUs was studied through adhesion and proliferation of RAW264.7 cells on PEG-ZSMPUs films. RAW264.7 cells were seeded on 0.2PEG-ZSMPU, 0.4PEG-ZSMPU, 0.6PEG-ZSMPU, 0.8PEG-ZSMPU and 1PEG-ZSMPU films, and then cultured at 37 °C for 24 h. Cell viability and proliferation were then evaluated. To investigate the cell adhesion and viability, the live/dead staining of RAW264.7 cells on different substrates was conducted. The results showed that most RAW264.7 cells were viable (indicated by green color stain) and displayed sphere-like shaped morphologies (Figure 11). These results demonstrated that PEG-ZSMPUs possessed a good biocompatibility while showed no effects on cell adhesion and morphology.

**Figure 12:**
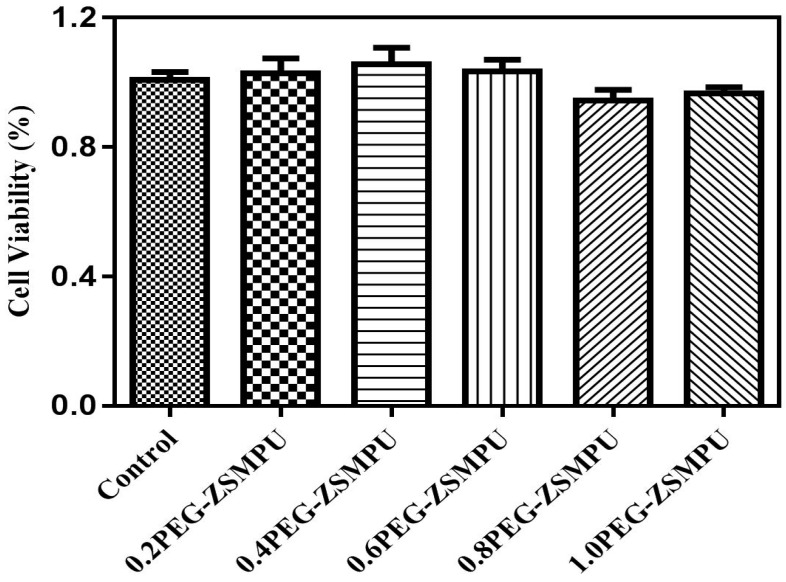
The cell activity of RAW264.7 cells incubated with the PEG-ZSMPUs films and Control sample.

In addition, the viability of treated RAW264.7 cells was studied using a tetrazolium salt (WST-8)-based colorimetric assay according to the Cell Counting Kit-8 (CCK-8) [20] and calculated by normalization in reference to the untreated cells [21]. The results shown in Figure 12, viability of RAW264.7 cells treated with PEG-ZSMPUs of different molar ratios of 1,3-PS and MDEA, demonstrated that more than 90% cell viability were observed in 0.2PEG-ZSMPU, 0.4PEG-ZSMPU and 0.6PEG-ZSMPU (Figure 12). Whereas, the higher molar ratios of 1,3-PS and MDEA slightly reduced cell viability, as indicated by cell viabilities of 0.8PEG-ZSMPU and 1PEG-ZSMPU, which were slightly lower than the control. Analysis of results above demonstrated that all of the PEG-ZSMPUs have good biocompatibility, and this is highly consistent with our previous investigations on zwitterionic polyurethanes [21]. 

**Figure 13:**
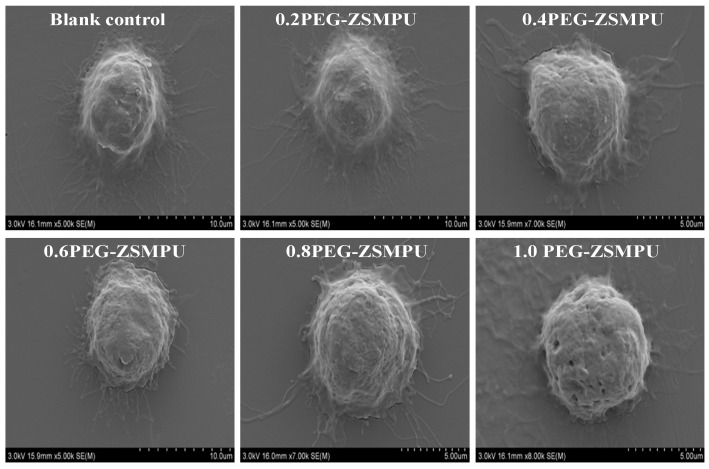
SEM images of RAW264.7 Cells incubated with PEG-ZSMPUs and the control sample.

Furthermore, the biocompatibility of polymers and bioactivity of treated RAW264.7 cells were also observed in SEM images. The SEM images of RAW264.7 cells treated with PEG-ZSMPUs are shown in Figure 13 demonstrated that cells had many clear filopodia so that they were able to adhere well to PEG-ZSMPUs substrate. Thus, it could be concluded that PEG-ZSMPUs have good biocompatibility. It has been shown that enhanced bioactivity of macrophages promotes phagocytosis of invasive particles, tumour cells and bacteria. On the other hand, weak bioactivity reduces the loss of drug carriers and implanted devices. Thus, PEG-ZSMPUs may have high potential application in smart biomedical fields [25].

## 4. Conclusions

In this work, zwitterionic PEG-ZSMPUs were synthesized using polyurethane polymerization from MDEA, HDI and PEG6000, followed by a ring-opening reaction with 1,3-PS. The structure and properties of zwitterionic PEG-ZSMPUs were systematically investigated. The results demonstrated that PEG-ZSMPUs formed phase separation structure that was composed of crystalline soft phase and amorphous hard phase. The zwitterionic hard segment greatly influenced their structures and properties. The MDEA-PS zwitterionic segments had a tendency to form ionic clusters in hard segments. Shape memory analysis showed that zwitterionic PEG-ZSMPUs had thermal-induced SMEs. 1.0PEG-ZSMPU had >78% and >94% of shape recovery and shape fixity, respectively. Finally, the cytotoxic assays demonstrated that MDEA-PS zwitterionic segment improved the biocompatibility of PEG-ZSMPUs. The zwitterionic PEG-ZSMPUs are thus expected to have promising application in smart biomedical fields.

## Figures and Tables

**Table 1:** Composition of Polyethylene glycol (PEG)-ZSMPUs.

Samples	Soft segment		Hard segment			R ^#^	ZHSC * (wt %)
	HDI (g)	PEG6000 (g)	HDI (g)	MDEA (g)	1,3-PS (g)		
0PEG-ZSMPU	0.33	11.67	4.68	3.32	0	0	40
0.2PEG-ZSMPU	0.33	11.67	4.68	3.32	0.68	0.2	42
0.4PEG-ZSMPU	0.33	11.67	4.68	3.32	1.36	0.4	44
0.6PEG-ZSMPU	0.33	11.67	4.68	3.32	2.04	0.6	46
0.8PEG-ZSMPU	0.33	11.67	4.68	3.32	2.72	0.8	47
1.0PEG-ZSMPU	0.33	11.67	4.68	3.32	3.40	1.0	49

^#^ R is the molar ratio of 1,3-propanesultone (1,3-PS) and methyldiethanolamine (MDEA); * ZHSC: zwitterionic hard segment content.

## Supplementary Materials

The following are available online at www.mdpi.com/2073-4360/9/10/465/s1, Figure S1: The ^1^HNMR spectra of 0PEG-ZSMPU and 0.8PEG-ZSMPU, Table S1: Elemental analysis results of PEG-ZSMPUs.
