# Supplementary material for: Shape Memory Polyurethanes Based on Zwitterionic Hard Segments
Source: Polymers (Basel). 2017 Sep 21;9(10):465. doi: 10.3390/polym9100465 (PMC6418923; doi:10.3390/polym9100465)
Supplement: Supplementary file 1 [file polymers-09-00465-s001.pdf]

polymers

Shape Memory Polyurethanes Based on Zwitterionic Hard Segments

Shuqin Fu[1,2,3], Huanhuan Ren[3], Zaochuan Ge[3], Haitao Zhuo[1,*] and Shaojun Chen[3,*]

1 Shenzhen Key Laboratory of Functional Polymer, College of Chemistry and Environmental Engineering, Shenzhen University, Shenzhen 518060, China; fusq@szu.edu.cn
2 Key Laboratory of Optoelectronic Devices and Systems of Ministry of Education and Guangdong Province, College of Optoelectronic Engineering, Shenzhen University, Shenzhen 518060, China;
3 Guangdong Research Center for Interfacial Engineering of Functional Materials, Shenzhen Key Laboratory of Polymer Science and Technology, Shenzhen Key Laboratory of Special Functional Materials, Nanshan District Key Lab for Biopolymers and Safety Evaluation, College of Materials Science and Engineering, Shenzhen University, Shenzhen 518060, China; rhh1171849722@163.com (H.R.); gezc@szu.edu.cn (Z.G.)
* Correspondence: haitaozhuo@163.com (H.Z.); chensj@szu.edu.cn (S.C.); Tel./Fax: +86-755-26534562 (S.C.)

Supporting informations

Table S1. Elemental analysis results of PEG-ZSMPUs

Samples	Elemental content in theory (wt%)	Elemental content obtained by EA (wt%)			
	S	S	N	C	H
0PS-SMPU	0	0.01	6.66	52.09	8.20
0.2PS-SMPU	0.85	0.82	6.52	52.12	8.19
0.4PS-SMPU	1.64	1.76	5.96	48.04	7.33
0.6PS-SMPU	2.46	2.38	6.01	49.71	7.96
0.8PS-SMPU	3.16	3.09	5.73	47.88	7.77
1PS-SMPU	3.81	3.41	6.04	48.77	7.79

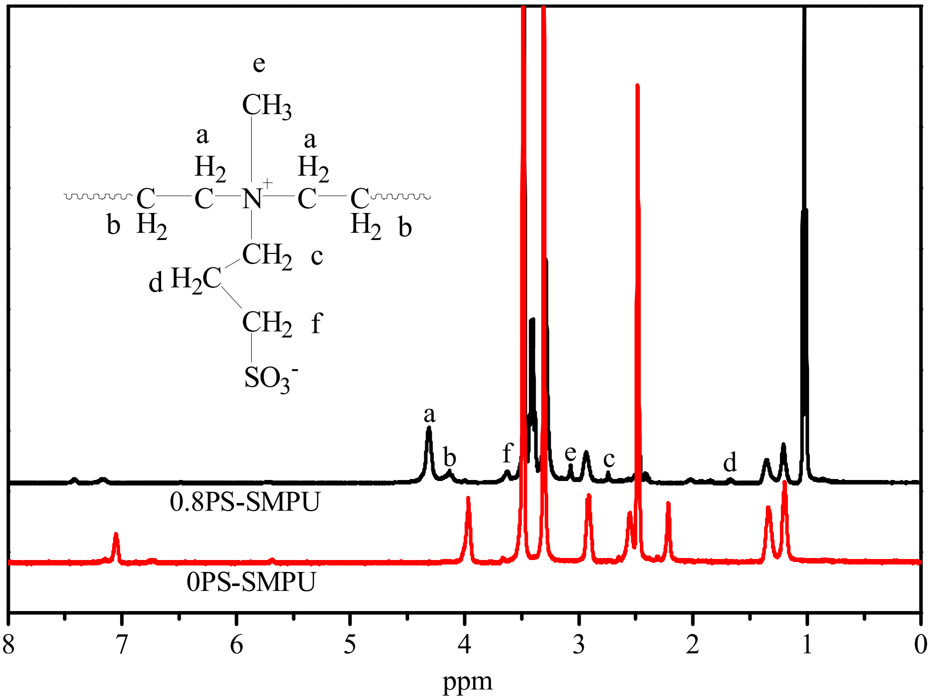

Figure S1. The ^{1}HNMR spectra of 0PS-SMPU and 0.8PS-SMPU
